# Maternal Dietary Patterns and Risk of Postpartum Depression: A Systematic Review

**DOI:** 10.1007/s10995-023-03781-7

**Published:** 2023-10-09

**Authors:** Yuyue Sun, Megan Ferguson, Marina M. Reeves, Jaimon T. Kelly

**Affiliations:** 1 School of Public Health, Faculty of Medicine, The University of Queensland, Brisbane, Australia; 2 Wellbeing and Preventable Chronic Diseases Division, Menzies School of Health Research, Charles Darwin University, Darwin, Australia; 3 Centre for Online Health, Faculty of Medicine, The University of Queensland, Brisbane, Australia; 4 Centre for Health Services Research, Faculty of Medicine, The University of Queensland, Brisbane, Australia

**Keywords:** Dietary patterns, Depression, Pregnancy, Postpartum, Maternal nutrition

## Abstract

**Objective:**

Postpartum depression (PPD) has deleterious effects on both maternal and child outcomes. Poor maternal nutrition during pregnancy has been implicated in the development of PPD. This review aimed to explore the association between the overall dietary intake patterns during pregnancy and the development of PPD.

**Methods:**

A literature search was performed in PubMed, Embase, Scopus, CINAHL, and PsycINFO databases for relevant randomized controlled trials, cohort and cross-sectional studies published up to 17th September 2020. Included studies assessed at least one dietary pattern during pregnancy and reported on PPD. The Newcastle Ottawa Scale and the Joanna Briggs Institute critical appraisal tools were used to assess the quality of methodology. A narrative analysis was conducted.

**Results:**

Ten studies (eight cohort and two cross-sectional) were included with substantial heterogeneity in measurements of dietary intake exposures and PPD. The studies identified several types of healthy dietary patterns, including a ‘healthy’, ‘health conscious’, ‘Japanese’, ‘high-glycemic index/glycemic load’, ‘Vegetable’, ‘Nut-Fruit’, ‘Seafood’, and ‘compliance with the Australian Dietary Guidelines’. The ‘Western’, ‘unhealthy’, ‘Beverage’, ‘Cereal-Meat’, and ‘Egg’ were labelled as unhealthy dietary patterns. Four of the eight studies showed an inverse association between adherence to healthy diets and risk of PPD, whereas only one of the seven studies showed that adherence to unhealthy diets was associated with increased risk of PPD. Methodological quality of the studies varied across the sample.

**Conclusions:**

Our findings indicate that adherence to a healthy diet may be beneficial for PPD. However, the relationship between unhealthy diets and PPD needs to be corroborated by more high-quality studies.

**Supplementary Information:**

The online version contains supplementary material available at 10.1007/s10995-023-03781-7.

## Significance

*What is already known about the topic?* Nutrition has been implicated in the development, prevention and management of depression during pregnancy. Individual nutrients and foods do not consider the inter-relations of the combined exposures of the dietary components. Thus, overall dietary pattern that better represents the way pregnant women eat has been proposed to be linked with PPD.

*What this study adds?* Healthy dietary patterns during pregnancy may be beneficial for PPD. However, the evidence suggests an inconclusive role of unhealthy dietary patterns and associations with PPD. Given the emerging evidence that supports a potential relationship between prenatal diet and maternal depressive symptoms, it is of utmost public health significance to continue research in this area.

**Supplementary Information:**

The online version contains supplementary material available at 10.1007/s10995-023-03781-7.

## Introductions

The postpartum period represents a highly vulnerable phase for the development of, or relapse of depressive events, in women. This may include feelings of sadness, anxiety, episodes of anger or rage, sleep disorder, social withdrawal, loss of interest, inability to bond with the child, and self-harm owing to the significant physiological, hormonal, emotional and psychosocial changes that occur (Biaggi et al., 2016; Parsons et al., 2012; Shrivastava et al., 2015).

Postpartum depression (PPD) is defined as major or minor episodes of depressive symptoms which arise within one year after delivery of a baby (Stewart et al., 2003). The World Health Organization (WHO) reports that 13% of women experience a mental illness, mainly depression, immediately after childbirth (World Health Organization, 2015). However, the prevalence rate is still underreported, especially in low-middle income countries (WHO, 2015).

A nutritious diet has been shown to prevent mental illness through its contribution to the biosynthesis and regulation of brain functions, neuronal membrane fluidity, synaptic plasticity and neurotransmitters (Rechenberg & Humphries, 2013). Previous evidence has found that deficiency of certain nutrients, such as ω-3 polyunsaturated fatty acid (PUFA), magnesium, zinc, vitamin D and B group vitamins, and lower intakes of foods, such as oily fish and vegetables were associated with a higher risk of PPD (Amini et al., 2019; Hogg-Kollars et al., 2011; Rechenberg & Humphries, 2013). However, studies investigating individual foods and nutrients do not consider the inter-relations of the combined exposures of the dietary components (Hu, 2002; Wirfält et al., 2013). Dietary patterns refer to the habitual consumption of a variety, quantity and combination of various food and beverages, representing the overall exposures to diet components, and may have stronger effects on health outcomes (Sánchez et al., 2017).

Dietary patterns have been linked to depression. Evidence from experimental studies have shown that the perinatal (during the period around birth) consumption of a Western dietary pattern characterized by high intakes of fat and branched-chain amino acids induced postnatal mood disorders and depression-like phenotype in mouse dams (Bolton et al., 2017). In addition, the mother–child ‘Rhea’ cohort study in Greece reported that compliance with a healthy dietary pattern characterized by greater consumption of fruit, vegetables, non-meat protein, fish and seafood, dairy products and olive oil during pregnancy was associated with a lower risk of PPD (Chatzi et al., 2011). To our knowledge, only one systematic review has examined the association between dietary patterns and perinatal anxiety and depression (PAAD) (Silva et al., 2019). However, this review only included three papers reporting on prenatal dietary patterns and PPD, which may not be sufficient to draw reliable conclusions on the association between diet indices and risk of PPD. Furthermore, as this study excluded studies using dietary indices to derive dietary patterns, it likely ignores other studies reporting on dietary patterns which could help make more reliable interpretations. Systematically evaluating the link between dietary patterns and PPD from the available literature could provide more comprehensive understanding of the role that diet has in the development of depression in postpartum women.

Therefore, the aim of this systematic review was to evaluate the current literature examining the associations between overall dietary patterns during pregnancy and the development of PPD.

## Methods

This study adhered to PRISMA (Preferred Reporting Items for Systematic Reviews and Meta-Analyses) (Moher et al., 2009).

### Eligibility Criteria and Exclusion Criteria

The eligibility criteria were: (1) any dietary patterns or the overall habitual dietary intake during pregnancy identified by study authors as the exposure, and where the primary outcome was incidence of PPD; (2) dietary patterns derived without statistical technique restriction (e.g. using ‘a posteriori’ approach or ‘a priori’ approach); (3) randomized controlled trial (RCT), cohort or cross-sectional study design; (4) full-text available; (5) studies published in English and peer-review journals; (6) participants over 18 years old.

The exclusion criteria were: (1) deviation from the topic of research (i.e. studies that examine antenatal depression or other psychiatric diseases as the primary outcome and that focus on the effects of individual nutrients, dietary components or food groups); (2) narrative articles without original data; (3) studies that utilized animal models; (4) adolescent mothers (as the diagnostic tools utilized to measure PPD in adolescent mothers compared to adult mothers are different) (Hymas & Girard, 2019).

### Search Strategy for Identification of Studies

A search was performed across five databases: PubMed, Embase, Scopus, CINAHL and PsycINFO, to identify relevant articles published to 17th September 2020. The search terms used across each database are available in Online Resource 1. Reference lists of eligible studies were manually searched to identify other potentially relevant studies for inclusion.

The search and title/abstract screening, and full text screening were independently performed by the first author (YS) and a research assistant to determine study eligibility with any disagreement resolved through discussion. If the study was unclear in meeting the inclusion criteria, the full-text was checked by the other two review authors (JK & MF).

Extracted data included study design, author, publication year, country, name of the study (where available), recruitment information, participant characteristics, diet measurement tools used to derive dietary patterns, identified dietary patterns, measurement tools to assess PPD, timing of assessment, results and additional details where relevant. A sub-set of included studies were discussed between all authors (YS, MF, JK & MR) to ensure the most appropriate data had been extracted.

### Methodological Quality

Methodological quality of each cohort study was assessed using the Newcastle Ottawa Scale (NOS) (Wells et al., 2019). The NOS includes three categories which are selection (four items), comparison (one item), and outcome (three items). For cross-sectional studies, the Joanna Briggs Institute (JBI) critical appraisal tool was used for quality assessment (The Joanna Biggs Institute, 2017). This tool includes eight items, such as study subjects, measurement and statistical analysis. Determining the risk of publication bias was not possible due to the small number of studies included. There was some nuance to assessing the assessment of exposure (dietary pattern) and outcome (PPD). For exposure, we graded this criterion a PY if the study used face-to-face structured interviews together with self-administered questionnaires, and a PN if it only used self-reports. Similarly for outcome, we graded a Y for studies using face-to-face structured interviews together with self-administered questionnaires, or a PY if the study utilized a maternal PPD self-report that is found to be validated and have a relatively high sensitivity and specificity against a mental health professional interview. We noted the reliability and validity and included this in our assessment.

### Data Analysis

Due to the heterogeneity of the study designs and diet exposures, a meta-analysis was not possible. Therefore, a narrative analysis of the results of the included studies was conducted, specifically of the results of the associations between exposures of dietary patterns and primary outcome of PPD. Given diverse definitions for dietary patterns with similar food components presented in studies, the healthy dietary pattern independent of the designation specified in the original study was defined as a diet consisting predominantly of vegetables, fruits, whole grains, plant-based protein, fish and shellfish, seafood products, dairy products and polyunsaturated fat. In contrast, the unhealthy dietary pattern was rich in red and processed meats, poultry, eggs, refined grains, confectionery, beverages, condiments, sodium, and high fat foods. Studies were grouped according to these two dietary pattern categories (i.e. ‘healthy’ and ‘non-healthy’). Where the diet exposures and primary outcome were reported as a ratio, a linear association was assumed, and the highest category of adherence to the lowest category of adherence to a dietary pattern was compared. Additionally, difference in the mean scores of the primary outcome across different levels of diet exposures was reported. For continuous data, the β-coefficient was used. The statistical significance was interpreted in two ways. Firstly, 95% confidence intervals (CIs) were used where the two intervals do not include the possibility of 1.00. Where the 95% CIs were not available, the reported p-values of the association (assuming a statistical significance of < 0.05) was utilized. Where neither 95% CIs or p-values were reported, a non-significant association was assumed.

## Results

### Study Selection

**Fig. 1:**
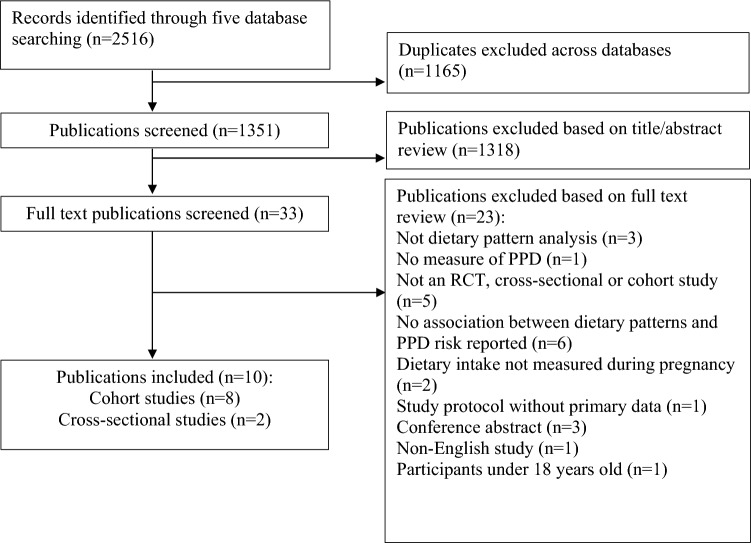
Preferred reporting items for systematic reviews and meta-analyses (PRISMA) flow chart of studies through review process

A total of 2516 articles were identified in the search. After removing duplicates (n = 1165), and excluding articles based on titles and abstracts (n = 1318), 33 full-text publications were screened. Twenty-three papers were further excluded for not meeting the inclusion criteria (Fig. 1), leaving ten publications included in the systematic review.

### Study Characteristics

A summary of study characteristics is presented in Table 1. Of the ten included studies, there were eight cohort studies and two cross-sectional studies. Two studies were conducted in Australia (Baskin et al., 2017; Huddy et al., 2016), Japan (Murakami et al., 2008; Okubo et al., 2011), the United Kingdom (UK) (Barker et al., 2013; Pina-Camacho et al., 2015), and one study in Austria (Hogg-Kollars et al., 2011), China (Cao et al., 2019), France (Galera et al., 2018), and Greece (Chatzi et al., 2011).

**Table 1:** Characteristics of the studies included in the systematic review (n = 10)

Study	Participants	Exposure	Outcome, covariates	Results
Cohort studies				
OMCHS Okubo et al. (2011) Japan	Recruited from 2001 to 2003 Neyagawa city & nine other municipalities N = 865 Age (yrs): 29.9 ± 4.0 BMI (kg/m^2^): 21.5 ± 2.8 Parity of 1 or more (%): 50.9 Gestation (wks): 18.0 ± 6.8	DHQ recalled 1 month prior from 20th week of gestation Dietary pattern derived using factor analysis Healthy^a^ Western^a^ Japanese^a^	Japanese EPDS, measured 2–9 months postpartum EPDS cut-off point: ≥ 9 Attrition rate: 0 Multivariable adjustment: Age, parity, cigarette smoking, family income, occupation, education, BMI, medical problems in pregnancy & others	Prevalence of PPD: 14% Healthy—PPD (OR and 95%CI): Q4 compared to Q1 (ref): 0.94 (0.52, 1.69) Western—PPD (OR and 95%CI): Q4 compared to Q1 (ref): 0.73 (0.42, 1.24) Japanese—PPD (OR and 95%CI): Q4 compared to Q1 (ref): 0.96 (0.56, 1.64)
The mother–child ‘Rhea’ cohort study Chatzi et al., 2011 Greece	Recruited from 2007 to 2008 Prefecture of Heraklion, Crete N = 529 Age (yrs): NR BMI (kg/m^2^): NR	FFQ recalled over pregnancy from mid-pregnancy (14–18th week of gestation) Dietary pattern derived using principle component analysis Health conscious^a^ Western^a^	EPDS, measured 8–10 weeks postpartum EPDS cut-off point: linear High level of EPDS cut-off point: ≥ 13 Attrition rate: 0 Multivariable adjustment: age, ethnic origin, education, smoking during pregnancy, PPD in previous pregnancies, total energy intake during pregnancy & others	Prevalence of PPD: 14% Health conscious—EPDS (β-Coefficient and 95%CI): T3 compared to T1 (ref): − 1.75 (− 3.22, − 0.28) Western—EPDS (β-Coefficient and 95%CI): T3 compared to T1 (ref): 1.32 (− 0.19, 2.76) Health conscious—high levels of PPD (RR and 95%CI): T3 compared to T1 (ref): 0.51 (0.25, 1.05) Western—high levels of PPD (RR and 95%CI): T3 compared to T1 (ref): 1.14 (0.58, 2.26)
Baskin et al. (2017) Australia	Recruited from 2010 to 2011 Through advertisements in online forums, obstetric officers and parenting magazines N = 167 Age (yrs): 30.55 (± 4.24) BMI (kg/m^2^): 25.44 ± 4.98 ^†^ Primiparous (%): 54.9^b^ Gestation (wks): 10–16	FFQ recalled 3 months prior from T1 (16.70th ± 0.91th week of gestation) and from T2 (32.89 ± 0.89th week of gestation) Dietary pattern derived using principle component analysis Healthy^a^ Unhealthy^a^	EPDS, measured 13.51 ± 1.97 weeks postpartum EPDS cut-off point: linear Attrition rate: 0 Multivariable adjustment: age, pre-pregnancy BMI, education, income, parity, history of depression, exercise & others	Prevalence of PPD: NR Healthy (T1)—PPD (β-Coefficient and p value): 0.03, NS Healthy (T2)—PPD (β-Coefficient and p value): 0.05, NS Unhealthy (T1)—PPD (β-Coefficient and p value): 0.12, NS Unhealthy (T2)—PPD (β-Coefficient and p value): -0.14, NS
OMCHS Murakami et al. (2008) Japan ALSPAC Barker et al. (2013) UK	Recruited from 2001 to 2003 Neyagawa city & nine other municipalities N = 865 Age (yrs): 29.9 ± 4.0 BMI (kg/m^2^): 21.5 ± 2.8 Parity of 1 or more (%): 50.9 Gestation (wks): 18.0 ± 6.8 Recruited from 1991 to 1992 The former Avon Health Authority in the south west of England N = 6979 Age (yrs): NR BMI (kg/m^2^): NR Primiparity (%): 54.9 Gestation (wks): 18 Ethnicity (%): 94.4 white	DHQ recalled 1 month prior from 20th week of gestation Dietary pattern derived using Standard Tables of Food Composition A high-GI diet A high-GL diet FFQ recalled 7 days prior from 32th week of gestation Dietary pattern derived using factor analysis Healthy^a^ Unhealthy^a^	Japanese EPDS, measured 2–9 months postpartum EPDS cut-off point: ≥ 9 Attrition rate: 0 Multivariable adjustment: Age, parity, cigarette smoking, family income, occupation, education, BMI, medical problems in pregnancy & others EPDS, mean of the scores at 8 weeks, 8 months, 21 months and 33 months EPDS cut-off point: linear Attrition rate: 0 Multivariable adjustment: Parity, education, marital status, birth complications, substance use & mother experiencing cruelty from partner	Prevalence of PPD: 14% Dietary GI—PPD (OR and 95%CI): Q4 compared to Q1 (ref): 0.72 (0.41, 1.26) Dietary GL—PPD (OR and 95%CI): Q4 compared to Q1 (ref): 0.63 (0.31, 1.31) Prevalence of PPD: NR Unhealthy—PPD (β-Coefficient and p value) 0.10, < 0.05 Healthy—PPD (β-Coefficient and p value) − 0.03, < 0.05
ALSPAC Pina-Camacho et al. (2015) UK Cao et al., (2019) China EDEN Galera (2018) France	Recruited from 1991 to 1992 The former Avon Health Authority in the south west of England N = 7814 Age (yrs): 25.39 ± 4.86 ^b^ BMI (kg/m^2^): NR Primiparity (%): 34.9 Gestation (wks): 18 Ethnicity (%): 95.0 white Recruited from 2015 to 2017 Two maternal and child health centers in Tianjin, China N = 1659 Age (yrs): 31.07 ± 3.91; ^c^ 30.80 ± 3.71^d^ BMI(kg/m^2^): NR Gestational (wks): 6.1% < 37; ^c^ 4.9% < 37 ^d^ Primiparity (%): 68.0; ^c^ 72.1^d^ Recruited from 2003 to 2005 Two French university hospitals (Nancy and Poitiers) N = 1242 Age (yrs): 29.3 ± 4.7 Pre-pregnancy BMI(kg/m^2^): 23.1 ± 4.3 Multiparity (%): 53.0	FFQ recalled 7 days prior from 32^th^ week of gestation Dietary pattern derived using factor analysis Unhealthy ^a^ FFQ recalled during the third trimester Dietary pattern derived using principal component analysis Beverage^a^ Vegetable^a^ Cereal-Meat^a^ Nut-Fruit^a^ Egg^a^ Seafood^a^ FFQ recalled over the last trimester of pregnancy Dietary pattern derived using principal component analysis Healthy^a^ Western^a^	EPDS, mean of the scores at 8 weeks, 8 months, 21 months and 33 months EPDS cut-off point: linear Attrition rate: 0 Multivariable adjustment: Parity, education, marital status, birth complications, substance use mother experiencing cruelty from partner & others Chinese SDS, measured 6–12 weeks postpartum SDS cut-off point: ≥ 50 Attrition rate: 0 Multivariable adjustment: Age, family monthly income, education, gestational age at delivery, delivery pattern, parity & living with parents CES-D, measured 12 months postpartum CES-D cut-off point: NR Attrition rate: 6.5% Multivariate adjustment: Education, age, smoking pre-pregnancy BMI, alcohol intake, gestational diabetes, maternal anxiety, multiparity & others	Prevalence of PPD: NR Unhealthy—PPD (β-Coefficient and p value) − 0.03, > 0.05 Prevalence of PPD: 34% Beverage—PPD (OR and 95%CI): T3 compared to T1 (ref): 1.00 (0.77, 1.30) Vegetable—PPD (OR and 95%CI): T3 compared to T1 (ref): 1.05 (0.82, 1.36) Cereal-Meat—PPD (OR and 95%CI): T3 compared to T1 (ref): 1.10 (0.85, 1.43) Nut-Fruit—PPD (OR and 95%CI): T3 compared to T1 (ref): 0.74 (0.57, 0.96) Egg—PPD (OR and 95%CI): T3 compared to T1 (ref): 1.18 (0.91, 1.52) Seafood—PPD (OR and 95%CI): T3 compared to T1 (ref): 0.75 (0.58, 0.98) Prevalence of PPD: NR Healthy (lowest quartile)—PPD (mean score ± SD and p value) 4.4 ± 4.4, 0.98 Healthy (others)—PPD (mean score ± SD and p value) 4.4 ± 4.7, 0.98 Western (highest)—PPD (mean score ± SD and p value) 4.8 ± 5.0, 0.09 Western (others)—PPD (mean score ± SD and p value) 4.3 ± 4.5, 0.09
Cross-sectional studies				
InFANT Extend Huddy (2016) Australia	Recruited from 2010–2011 Melbourne and Geelong regions of Victoria Australia N = 437 Age (yrs): 32.1 ± 4.3 BMI (kg/m^2^): 26.3 ± 5.0 Parity of 1 or more (%): NR Gestation (wks): NR	FFQ recalled 12 months prior from 3 months postpartum Dietary pattern derived using Dietary Guideline Index score^a^	CES-D, measured 3 months postpartum CES-D cut-off point: ≥ 10 Multivariable adjustment: age, BMI, education, smoking, television viewing and sleep quality & others	Prevalence of PPD: 35.2% Dietary Guideline Index—PPD (β-Coefficient and 95%CI): − 0.034 (− 0.056, − 0.012)
Hogg-Kollars (2011) Austria	Recruited from 2003 to 2008 Through e-mail announcement to parent-toddler related discussion groups in Austria and via snowball sampling N = 400 Age (yrs): NR BMI (kg/m^2^): NR Parity of 1 or more (%): NR Gestation (wks): NR	FFQ recalled over pregnancy at retrospective survey Method to derive dietary pattern: NR Vegetarian diet Omnivorous diet	Self-report PPD, measured at retrospective survey PPD cut-off point: NR	Prevalence of PPD: 20.8% Vegetarian—depression (n and %): Non depressed: 10, 52.6 Depressed: 9, 47.4 Total: 19, 100 Omnivorous—depression (n and %): Non depressed: 307, 80.6 Depressed: 74, 19.4 Total: 381, 100 Chi-square (Pearson) = 8.6; df = 1, p = 0.003

*OMCHS* Osaka maternal and child health study; *yr(s)* year (s); *BMI* body mass index; *wk(s)* week(s); *DHQ* dietary history questionnaire; *EPDS* edinburgh postnatal depression scale; *PPD* postpartum depression; *OR* odds ratio; *CI* confidence interval; *ref* reference; *NR* not reported; *FFQ* food frequency questionnaire; *RR* relative risk; *NS* non-significant; *CES-D* centre for epidemiological studies depression scale; *SDS* self-rating depression scale; *EDEN* Etude des Déterminants pré‐ et postnatals précoces du développement et de la santé de l'Enfant; InFANT, Melbourne Infant Feeding, Activity and Nutrition Trial; *GI* glycemic index; *GL* glycemic load; *ALSPAC* avon longitudinal study of children and parents

^a^See Table 2

^b^A certain proportion of women with available information from the total selected sample

^c^1090 women with < 50 self-rating depression scale scores and diagnosed with no depression

^d^569 women with ≥ 50 self-rating depression scale scores and diagnosed with depression

### Dietary Intake Assessment

Food Frequency Questionnaire (FFQ) and Dietary History Questionnaire (DHQ) were the methods used to measure participants’ habitual diet intake. All but one study (Hogg-Kollars et al., 2011) reported the method of deriving dietary patterns; seven studies (Barker et al., 2013; Baskin et al., 2017; Cao et al., 2019; Chatzi et al., 2011; Galera et al., 2018; Okubo et al., 2011; Pina-Camacho et al., 2015) used either common factor analysis or principle component analysis, considered ‘a posteriori’ approach (Hu, 2002), to derive a dietary pattern through statistical modelling of dietary data collected from the sample population. One study (Huddy et al., 2016) used the Dietary Guideline Index, considered ‘a priori’ approach (Hu, 2002), to measure the degree to which a subject’s habitual diet complies with the indicators identified for each dietary guideline with the development of the sex- and age-specific cut-offs and serving recommendations for major food groups according to the 2013 Australian Dietary Guidelines (ADGs). One study (Murakami et al., 2008) assessed dietary glycemic index (GI) and glycemic load (GL) using the Standard Tables of Food Composition in Japan.

The studies identified several types of dietary patterns. The characteristics of these dietary patterns are presented in Table 2. The ‘healthy’, ‘health conscious’, ‘Japanese’, ‘high-GI/GL’, ‘Vegetable’, ‘Nut-Fruit’, ‘Seafood’, and ‘compliance with the ADGs’ were labelled as healthy dietary patterns due to the foods they comprise being aligned with the definition of healthy diets and having potential protective roles against PPD. In contrast, the ‘Western’, ‘unhealthy’, ‘Beverage’, ‘Cereal-Meat’, and ‘Egg’ were labelled as unhealthy dietary patterns. The ‘vegetarian’ and ‘omnivorous’ diets were not grouped into either healthy dietary pattern or unhealthy dietary pattern owing to the lack of an explicit definition of the pattern characteristics (Hogg-Kollars et al., 2011).

**Table 2:** Characteristics of dietary patterns

Study	Type of diet	Foods used to define pattern
OMCHS Okubo et al (2011) Japan	Healthy Western	White vegetables, potatoes, green and yellow vegetables, seaweeds, fruit, sea products, fish and shellfish Beef and pork, chicken, vegetable oil, processed meat, eggs and salt-containing seasonings Japanese: miso soup, pickled vegetables, rice, fish and sea products
The mother–child ‘Rhea’ cohort study Chatzi et al., (2011) Greece	Health Conscious Western	Fruit, vegetables, pulses, nuts, fish and seafood, dairy products and olive oil Meat and meat products, cereals, potatoes, sugar and sweets, fats except olive oil, salty snacks, eggs, beverages and sauces
Baskin et al (2017) Australia	Healthy Unhealthy	Vegetables, eggs, nuts, fruit, whole grains, tea, water, fish and seafood Refined grains, condiments, fast foods, high-energy drinks, hot chips, sweets and desserts, fruit juice, red meats, high-fat dairy, nuts-based and oil/vinegar-based dressing
ALSPAC Barker et al (2013) UK	Healthy Unhealthy	Fish (i.e. oily fish and white fish), non-meat protein (i.e. nuts and pulses), vegetables (i.e. green vegetables, carrots, cabbage, other root vegetables) Processed food (i.e. meat pies or pastries, fried food, crisps, chips), junk food (i.e. cakes/buns, chocolate bars, biscuits)
ALSPAC Pina-Camacho e tal (2015)	Unhealthy	Processed food (i.e. meat pies or pastries, fried food, chips), confectionery (i.e. crisps, cakes or buns, chocolate bars, biscuits)
UK Cao et al., (2019) China	Beverage Vegetable Cereal-Meat Nut-Fruit Egg Seafood	Pickled smoked products, alcohol, beverages and desserts Vegetables, tubers and legumes Cereals, milk and milk products, poultry and meat Nuts and fruits Eggs Seafood
EDEN Galera et al (2018) France	Healthy Western	Vegetables, fruit, fish, and whole grain cereals Processed and snacking foods
Huddy et al (2016) Australia	The dietary Guideline Index	Diet variety, vegetables, grains and cereals, fruit, dairy and alternatives, meat and alternatives, discretionary food items, fat and sugar, and alcohol intakes

### Mental Health Assessment

Evaluation of the PPD of the participants was conducted by the researchers with three different instruments across all the studies. The Edinburgh Postnatal Depression Scale (EPDS) was used by the majority of studies (Barker et al., 2013; Baskin et al., 2017; Chatzi et al., 2011; Murakami et al., 2008; Okubo et al., 2011; Pina-Camacho et al., 2015). Centre for Epidemiological Studies Depression Scale (CES-D) was used by two studies (Galera et al., 2018; Huddy et al., 2016), one study used the Chinese version of the Self-Rating Depression Scale (SDS) (Cao et al., 2019), and one study did not objectively measure PPD in participants, rather utilized an online self-reported mechanism (Hogg-Kollars et al., 2011). Six studies reported on the proportion of the sample classified as PPD, ranging from 14% to 35.2% (Cao et al., 2019; Chatzi et al., 2011; Hogg-Kollars et al., 2011; Huddy et al., 2016; Murakami et al., 2008; Okubo et al., 2011).

### Associations Between Dietary Patterns and PPD

Four of the eight studies (50%) examining the associations of healthy diets reported a significant association to PPD. In the ‘Rhea’ mother–child cohort, higher compliance with a ‘health conscious’ diet over pregnancy was significantly associated with lower risk of PPD between eight and ten weeks postpartum (highest exposure *vs.* lowest exposure: β-coefficient = − 1.75, 95% CI − 3.22, − 0.28) when analysis conducted with the PPD score as a continuous outcome variable (Chatzi et al., 2011). In the study conducted by Barker et al. (2013), a ‘healthy’ dietary pattern at 32 weeks gestation had a small but significant effect on decreased PPD between eight weeks and 33 months postpartum (β-coefficient = − 0.03, p < 0.05). The study performed by Cao et al. (2019), indicated that greater adherence to the ‘Nut-Fruit’ and ‘Seafood’ dietary patterns during the third trimester was significantly associated with a lower risk of PPD (highest exposure *vs.* lowest exposure: OR = 0.74, 95%CI 0.57, 0.96; T3 *vs.* T1: OR = 0.75, 95%CI 0.58, 0.98). Women, who had ‘higher compliance with the ADGs’ over pregnancy were less likely to develop PPD three months postpartum (β-coefficient = − 0.034; 95% CI -0.056 to − 0.012) (Huddy et al., 2016).

In the OMCHS cohorts, the ‘healthy’, ‘Japanese’, ‘high-GI’, and ‘high-GL’ diets at 20 weeks gestation were not found to be associated with the risk of depression between two and nine months postpartum (Murakami et al., 2008; Okubo et al., 2011). The study performed by Baskin et al. (2017) and the study conducted by Galera et al. (2018) showed that there was no significant relationship between the ‘healthy’ diet during pregnancy and PPD.

However, one (14%) (Barker et al., 2013) of the seven studies examining the associations of unhealthy dietary patterns and PPD reported that an ‘unhealthy’ dietary pattern at 32 weeks gestation was significantly associated with increased risk of PPD between eight weeks and 33 months postpartum (β-coefficient = 0.10, p < 0.05). Three studies reported that there was no definitive evidence of a relationship between the ‘Western’ dietary pattern during pregnancy and the incidence of PPD ranging from eight weeks to 12 months postpartum (Chatzi et al., 2011; Galera et al., 2018; Okubo et al., 2011). Two studies found no significant association between the ‘unhealthy’ dietary pattern at around 17 and 33 weeks gestation and PPD between eight weeks and 33 months postpartum (Baskin et al., 2017; Pina-Camacho et al., 2015). The study performed by Cao et al. (2019) reported that no association was observed between the ‘Beverage’, ‘Cereal-Meat’, and ‘Egg’ patterns during the third trimester and risk of PPD between six and 12 weeks postpartum.

The study conducted by Hogg-Kollars et al. (2011) reported on vegetarian and omnivore diets which were not able to be categorized as either healthy or unhealthy dietary pattern. The study found that vegetarians (47.4%) were more depressed compared to omnivores (19.4%).

### Critique of Methodological Quality

Methodological quality of the studies varied across the sample. As shown in Table 3, the ascertainment of exposure and outcome in the majority of the cohort studies were rated as a high risk of bias as self-report tools were utilized rather than structured interviews or physician-made diagnoses of the outcome. A small portion of the included studies did not report whether the outcomes of interest had been present at the baseline, whereas some retrospective cohort studies had a statement of maternal depression at the time of recruitment. The length of follow-up was adequate in all the cohort studies as the normal range of onset of PPD is between one and 12 months postpartum and the minimum onset of PPD is two weeks postpartum.

**Table 3:** Quality assessment of studies for cohort studies

Study	Selection				Comparability	Outcome		
	Representativeness of exposed cohort	Selection of non-exposed cohort	Ascertainment of exposure	Demonstration outcome of interest not present at start of study	Comparability of cohort on basis of design or analysis	Assessment of outcome	Long enough follow-up for outcome to occur	Adequacy of follow-up of cohorts
Okubo (2011)	Y	Y	PY Validated, self-administered, comprehensive DHQ	NI	PY	PY Self-administered EPDS	Y	Y
Chatzi et al., (2011)	Y	Y	Y Face-to-face structured questionnaires together with self-administered questionnaires	Y	Y	Y Face-to-face structured questionnaires, self-administered questionnaires medical records	Y	Y
Baskin (2017)	PY	Y	PY Validated, self-administered, FFQ	N	Y	PY Validated, self-rating EPDS	Y	Y
Murakami (2008)	Y	Y	PY Validated, self-administered, comprehensive FFQ	NI	PY	PY Self-administered EPDS	Y	Y
Barker (2013)	PY	Y	PY Self-completed FFQ	N	PY	PY Validated, self-report EPDS	Y	Y
Pina-Camacho (2015)	PY	Y	PY Self-completed FFQ	N	PY	PY Validated, self-report EPDS	Y	Y
Cao et al., (2019)	PY	Y	PY FFQ administered through face-to-face interviews	N	PY	PN Self-rating depression scales	Y	Y
Galera (2018)	PY	Y	PY Validated, self-administered FFQ	N	Y	PY Self-administered EPDS	Y	PY

Wells et al., (2019)

*Y* yes; *PY* probably yes; *PN* probably no; *N* no, *NI* no information

As shown in Table 4, the reliability of the exposure tools was rated as ‘unclear’ and ‘no’ for the two cross-sectional studies. In addition, one study (Hogg-Kollars et al., 2011), had a high risk of bias in five of the eight domains due to the absence of considerations concerning any potential covariates, absence of validated and reliable tools assess both dietary intake and PPD, and unclear statistical analysis technique.

**Table 4:** Quality assessment of studies for cross-sectional studies

Study	Inclusion criteria clearly defined	Study subjects and setting described in detail	Valid and reliable measurement of exposure	Objective, standard criteria used for measurement of condition	Confounding factors identified	Strategies for dealing with confounding factors stated	Valid and reliable measurement of outcomes	Appropriate statistical analysis
Huddy et al (2016)	Yes	Yes	Yes, Unclear Validated, self-administered FFQ; no mention of repeatability of measurements of dietary intakes	Yes	Yes	Yes	Yes Self-administered questionnaire and anthropometric data	Yes
Hogg-Kollars et al (2011)	Yes	Yes	No Self-developed 55-item non quantitative FFQ	Yes	No	No	No Absence of validated and reliable tools assess PPD	Unclear

The Joanna Biggs Institute. (2017)

## Discussion

The finding of this systematic review implies that healthy dietary patterns during pregnancy that include vegetables, fruits, nuts, pulses, fish and shellfish, seafood products, dairy products and olive oil, may be beneficial for PPD. However, it is important to note that there was inconsistency in the findings, and there is currently substantial heterogeneity in the literature which makes determining a potential relationship between dietary patterns and PPD difficult.

The results of the associations between healthy diets and PPD appear to be consistent with previous evidence showing components of a healthy diet may associate with PPD. For example, higher intake (> 40 g/d) of olive oil (a component of a healthy diet) assessed during pregnancy has been found to be significantly inversely associated with high levels of PPD symptoms (Chatzi et al., 2011). The Japan Environment and Children’s Study also found that the median intake of fish during pregnancy lying within the second to fourth quintile is significantly associated with reductions of PPD. This indicates that higher intake of fish may be protective against PPD (Hamazaki et al., 2020), which was a key component of the healthy dietary patterns.

PPD is one of the most prevalent complications of pregnancy and the postnatal period. The results of this review and future studies examining dietary patterns and maternal health have important public health implications. For example, the Healthy People 2030 concentrates on reducing pregnancy complications and maternal deaths and promoting the well-being for women after delivery (USPSTF, US Preventive Services Task Force, 2018). This framework includes maternal mental health as a high-priority public health issue, and one of the objectives that has developmental status is to increase the percentage of women who undergo screening for PPD at their postnatal checkups. The U.S. Preventive Services Task Force (USPSTF) recommends counseling interventions for PPD for women who have higher risk factors after delivery, and have a history or symptoms of depression (USPSTF, 2018). The results of this review can assist practitioners and public health workers advise expectant and planning mothers of diets better associated with improved mental health in the post-partum period.

Putative mechanisms for this potential association of unhealthy diets and the increased risk of PPD likely involve adiposity and inflammation. Research into the role of inflammation and development of depressive symptoms has recognized that sources of inflammatory processes, including cytokines, chemokines, and adipocytes might trigger the development of depression (Shelton & Miller, 2011).

Notwithstanding the potential for healthy diet patterns in reducing the prevalence of PPD, the evidence from this systematic review suggests an inconclusive role of unhealthy dietary patterns and associations with PPD. The non-significant association between unhealthy maternal dietary patterns and PPD could be explained by the short-term assessment of dietary intake during pregnancy and the heterogeneity of the reported studies. In the majority (80%) of the included studies, assessment of dietary intake was based on recent diet, namely the preceding seven days, one month or three months before completing the questionnaires. It is important however, to assess dietary intake over a longer period of time as eating behaviors often change before, during, and after pregnancy (Czech-Szczapa et al., 2015; Soares et al., 2009). During pregnancy, motivation for healthy eating habits may change relative to the pre-pregnant state as some women consider the benefit of child development and better prognosis for delivery and postnatal outcomes (Czech-Szczapa et al., 2015; Forbes et al., 2018). Equally, some women may experience (mostly) unhealthy cravings due to the physiological changes accompanying gestation, and may start their pregnancy adhering to unhealthy dietary patterns (Orloff & Hormes, 2014; Bodnar et al. 2017). Pregnancy complications such as gestational diabetes and gestational hypertension could also play a role in changes in dietary behaviors and mediate the association of diet and PPD. An expectant mother could for example, change her diet during pregnancy, then revert to her pre-pregnancy diet, or completely change her dietary intake pattern again up to 12 months post-partum, with each of these periods potentially impacting on PPD. Therefore, it is possible that associations of dietary patterns (which reflect a long-term dietary habit) to postpartum outcomes may not be evident within the short dietary assessment periods of the included studies. In order to detect the impact of dietary patterns, it is more effective to monitor over many years, as has been conducted in prior research (Francis et al., 2019; Khosravi et al., 2015). Although the timing of outcome was within the recognized periods of PPD, it is important to acknowledge the limitations of included studies regarding timing of the exposure assessment.

The other likely reason for the inconsistent findings among studies in this review is the heterogeneity observed across the studies. This includes heterogeneous study methods and definitions of ‘healthy’ and ‘unhealthy’ dietary patterns. As recognized earlier, eating behaviors can change during pregnancy, but the majority of the included studies measured dietary intake at a single time point rather than repeated measurements. With regard to the methods to derive overall dietary intake, statistically derived dietary patterns include all possible dietary exposures and represent actual eating behaviors of the individuals. Although the dietary index approach reflects compliance with serving and specific dietary recommendations from prevailing dietary guidelines, it may not wholly represent actual eating behaviors and the best available scientific evidence. In addition, multiple factors that influence the development of depression may have contributed to non-significant associations. Further, these studies were conducted in seven different countries, with cultural differences in food intake likely reflecting in differences in healthy and unhealthy dietary patterns between studies.

Given the epidemiological nature of the included studies, PPD was assessed using self-reported scales rather than structured interviews or physician’s diagnosis. The EPDS, a well-validated scale to assess PPD, was most commonly used by the majority of studies. Timing of assessing PPD varied across the cohort studies and ranged from eight weeks to 33 months postpartum. However, screening in the postnatal period indicating the risk of PPD does not reflect the actual onset of depressive symptoms. The symptoms may have occurred or even changed prior to or during pregnancy. While there are some women that develop PPD without pre-pregnant symptomatology, there are many women that have previous experience with anxiety or depression prior to pregnancy. Complications in pregnancy and stressful life events have both predicted an increase in PPD that are tied to the perinatal period (Baskin et al., 2017). There were also differences in the use of the EPDS across the studies to form a categorical or continuous variable outcome for PPD, with only one study reporting both (Chatzi et al., 2011). Furthermore, the majority of studies utilized self-administered FFQs or DHQs without face-to-face structured interviews to obtain dietary data. Although the validity and reliability of the questionnaires have been evaluated, the possibility of recall bias, social desirability reporting bias and non-differential misclassification might have existed in dietary exposures. For example, subjects might have reported healthier dietary intakes than what they actually ate. Therefore, the results were different even though the sample was the same. This variation in the instruments used may have added further heterogeneity to the studies as well as diluting and obscuring potentially significant associations between diet and PPD.

Our review highlights the paucity of studies evaluating dietary patterns and PPD. We believe our comprehensive search and thorough analysis of the available data to be a particular strength of this paper. However, several limitations need to be acknowledged. Firstly, there is a small pool of literature to draw from and the high heterogeneity as discussed above makes it difficult to draw reliable conclusions. Secondly, this review is based on observational evidence, and residual confounding cannot be completely ruled out. Third, there is a large variation regarding the use of statistical methods and level of detail reported as several studies lacked adequate statistical reporting including standard errors, p-values or 95% CIs. Fourth, there were not enough studies or information reported to determine the differences in in PPD according to socio-demographics and disparities. Finally, our findings might have been affected by reporting bias as we restricted to publications in English only.

The potential protective role of healthy diets can be conveyed to persons willing to prevent PPD onset. However, there is currently substantial heterogeneity in the literature which is predominantly of poor methodological quality. Therefore, more research using well-designed, high-quality methods is needed to determine the effect of dietary patterns on PPD before making public health recommendations. There is a greater need for well-designed, longitudinal studies informed by standard definitions and measurements of dietary patterns and PPD. Future studies that assess dietary intake at more than one single point of time prior to, during and after pregnancy are recommended.

## Conclusion

This review aimed to evaluate the potential association between dietary patterns during pregnancy and the development of PPD. Healthy dietary patterns during pregnancy may be beneficial for PPD. However, the relationship between unhealthy dietary patterns and PPD needs to be corroborated by more high-quality studies. Given the emerging evidence that supports a potential relationship between prenatal diet and maternal depressive symptoms, it is of utmost public health significance to continue research in this area.

### Supplementary Information

Below is the link to the electronic supplementary material.

Supplementary file1 (PDF 71 kb)

## Data Availability

Not applicable.
