# Supplementary material for: Maternal Dietary Patterns and Risk of Postpartum Depression: A Systematic Review
Source: Matern Child Health J. 2023 Oct 9;27(12):2077–90. doi: 10.1007/s10995-023-03781-7 (PMC10618401; doi:10.1007/s10995-023-03781-7)
Supplement: Supplementary file 1 — Supplementary file1 (PDF 71 kb) [file 10995_2023_3781_MOESM1_ESM.pdf]

Supplemental Table 1: Database-specific search strategy and terms

PubMed search terms:

(((((((((((((((((nutrit*[tiab]) OR diet[tiab]) OR diets [tiab]) OR dieting [tiab]) OR dietary[tiab]) OR dietitian[tiab]) OR dietician[tiab]) OR food*[tiab]) OR dietetic*[tiab]) OR nutrient*[tiab]) OR "Diet"[Mesh]) OR "Dietetics"[Mesh]) OR "Nutrients"[Mesh]) OR "Food"[Mesh])) AND (((((((((((((pregnan*[tiab]) OR gestation*[tiab]) OR gravidity*[tiab]) OR trimester*[tiab]) OR antenatal[tiab]) OR ante-natal[tiab]) OR "ante natal"[tiab]) OR prenatal[tiab]) OR pre-natal[tiab]) OR "pre natal"[tiab]) OR "Pregnant Women"[Mesh]) OR "Pregnancy Trimesters"[Mesh]) OR "Pregnancy"[Mesh]))) AND (((((((postpartum[tiab] OR post-partum[tiab] OR "post partum"[tiab] OR postnatal[tiab] OR post-natal[tiab] OR "post natal"[tiab] OR puerperium[tiab])) OR "Postpartum Period"[Mesh:NoExp])) AND (((depress*[tiab]) OR "Depression"[Mesh]) OR "Depressive Disorder"[Mesh:NoExp]))) OR "Depression, Postpartum"[Mesh])

Embase search terms:

('postnatal depression'/exp OR (('puerperium'/exp OR postnatal:ab,ti OR 'post natal':ab,ti OR postpartum:ab,ti OR 'post partum':ab,ti OR puerperium:ab,ti) AND ('depression'/exp OR depress*:ab,ti))) AND ('diet'/exp OR 'dietetics education'/exp OR 'nutrient'/exp OR 'food'/exp OR nutrit*:ab,ti OR diet:ab,ti OR diets:ab,ti OR dieting:ab,ti OR dietary:ab,ti OR dietitian:ab,ti OR dietician:ab,ti OR food*:ab,ti OR dietetic*:ab,ti OR nutrient*:ab,ti) AND ('pregnancy'/exp OR 'pregnant woman'/exp OR pregnan*:ab,ti OR gestation*:ab,ti OR gravidity*:ab,ti OR trimester*:ab,ti OR antenatal:ab,ti OR 'ante natal':ab,ti OR prenatal:ab,ti OR 'pre natal':ab,ti)

Scopus search terms:

TITLE-ABS (pregnan* OR gestation* OR gravidity* OR trimester* OR antenatal OR "ante-natal" OR "ante natal" OR prenatal OR "pre-natal" OR "pre natal")) AND (TITLE-ABS(nutrit* OR diet OR diets OR dieting OR dietary OR dietition OR dietician OR food* OR dietetics OR dietetic OR nutrient*)) AND (TITLE-ABS("post partum" OR "post-partum" OR postpartum OR "post natal" OR "post-natal" OR postnatal OR puerperium) AND TITLE-ABS(depress*))

CINAHL search terms:

(MH "Pregnancy Trimesters+") OR (MH "Pregnancy+") OR (MH "Expectant Mothers") OR TI (pregnan* OR gestation* OR gravidity* OR trimester* OR antenatal OR "ante natal" OR ante-natal OR prenatal OR "pre natal" OR pre-natal) OR AB (pregnan* OR gestation* OR gravidity* OR trimester* OR antenatal OR "ante natal" OR ante-natal OR prenatal OR "pre natal" OR pre-natal) AND (MH "Diet+") OR (MH "Nutritional Requirements+") OR (MH "Dietetics") OR (MH "Research, Dietetics") OR (MH "Food+") OR (MH "Nutrients+") OR TI (nutrit* OR diet OR diets OR dieting OR dietary OR dietitian* OR dietician* OR food* OR dietetic OR dietetics OR nutrient*) OR AB (nutrit* OR diet OR diets OR dieting OR dietary OR dietitian* OR dietician* OR food* OR dietetic OR dietetics OR nutrient*) AND (MH "Depression, Postpartum") OR (MH "Postnatal Period") OR TI (postnatal OR postpartum OR "post natal" OR "post partum" OR "post-natal" OR "post-partum" OR puerperium) OR AB (postnatal OR postpartum OR "post natal" OR "post partum" OR "post-natal" OR "post-partum" OR puerperium) OR (MH "Depression") OR TI depress* OR AB depress*

PsycInfo search terms:

((((((IndexTermsFilt: ("Diets"))) OR ((IndexTermsFilt: ("Food"))))) OR ((((abstract: (nutrit*))) OR ((abstract: (diet))) OR ((abstract: (diets))) OR ((abstract: (dieting))) OR ((abstract: (dietary))) OR ((abstract: (dietitian))) OR ((abstract: (dietician))) OR ((abstract: (food*))) OR ((abstract: (dietetic))) OR ((abstract: (dietetics))) OR ((abstract: (nutrient*)))) OR (((title: (nutrit*))) OR ((title: (diet))) OR ((title: (diets))) OR ((title: (dieting))) OR ((title: (dietary))) OR ((title: (dietitian))) OR ((title: (dietician))) OR ((title: (food*))) OR ((title: (dietetic))) OR ((title: (dietetics))) OR ((title: (nutrient*)))))) AND (((((abstract: (pregnan*))) OR ((abstract: (gestation*))) OR ((abstract: (gravidity*))) OR ((abstract: (trimester*))) OR ((abstract: (antenatal))) OR ((abstract: (“ante-natal”))) OR ((abstract: ("ante natal"))) OR ((abstract: (prenatal))) OR ((abstract: (“pre-natal”))) OR ((abstract: ("pre natal")))) OR (((title: (pregnan*))) OR ((title: (gestation*))) OR ((title: (gravidity*))) OR ((title: (trimester*))) OR ((title: (antenatal))) OR ((title: (“ante-natal”))) OR ((title: ("ante natal"))) OR ((title: (prenatal))) OR ((title: (“pre-natal”))) OR ((title: ("pre natal"))))) OR ((((IndexTermsFilt: ("Pregnancy"))))))) AND ((((((((abstract: (depress*))))) OR ((((title: (depress*)))))) OR (((((IndexTermsFilt: ("Major Depression")))) OR (((IndexTermsFilt: ("Depression (Emotion)"))))))) AND ((((((abstract: (postnatal)))) OR (((abstract: ("post natal")))) OR (((abstract: (“post-natal”)))) OR (((abstract: (postpartum)))) OR (((abstract: (“post-partum”)))) OR (((abstract: ("post partum")))) OR (((abstract: (puerperium))))) OR ((((title: (postnatal)))) OR (((title: ("post natal")))) OR (((title: (“post-natal”)))) OR (((title: (postpartum)))) OR (((title: (“post-partum”)))) OR (((title: ("post partum")))) OR (((title: (puerperium)))))) OR (((((IndexTermsFilt: ("Postnatal Period")))))))) OR (((IndexTermsFilt: ("Postpartum Depression"))))) OR Any Field: ((((((IndexTermsFilt: ("Diets"))) OR ((IndexTermsFilt: ("Food"))))) OR ((((abstract: (nutrit*))) OR ((abstract: (diet))) OR ((abstract: (diets))) OR ((abstract: (dieting))) OR ((abstract: (dietary))) OR ((abstract: (dietitian))) OR ((abstract: (dietician))) OR ((abstract: (food*))) OR ((abstract: (dietetic))) OR ((abstract: (dietetics))) OR ((abstract: (nutrient*)))) OR (((title: (nutrit*))) OR ((title: (diet))) OR ((title: (diets))) OR ((title: (dieting))) OR ((title: (dietary))) OR ((title: (dietitian))) OR

((title: (dietician))) OR ((title: (food*))) OR ((title: (dietetic))) OR ((title: (dietetics))) OR ((title: (nutrient*)))))) AND (((((abstract: (pregnan*))) OR ((abstract: (gestation*))) OR ((abstract: (gravidity*))) OR ((abstract: (trimester*))) OR ((abstract: (antenatal))) OR ((abstract: (“ante-natal”))) OR ((abstract: ("ante natal"))) OR ((abstract: (prenatal))) OR ((abstract: (“pre-natal”))) OR ((abstract: ("pre natal")))) OR (((title: (pregnan*))) OR ((title: (gestation*))) OR ((title: (gravidity*))) OR ((title: (trimester*))) OR ((title: (antenatal))) OR ((title: (“ante-natal”))) OR ((title: ("ante natal"))) OR ((title: (prenatal))) OR ((title: (“pre-natal”))) OR ((title: ("pre natal"))))) OR ((((IndexTermsFilt: ("Pregnancy"))))))) AND ((((((((abstract: (depress*))))) OR ((((title: (depress*)))))) OR (((((IndexTermsFilt: ("Major Depression")))) OR (((IndexTermsFilt: ("Depression (Emotion)"))))))) AND ((((((abstract: (postnatal)))) OR (((abstract: ("post natal")))) OR (((abstract: (“post-natal”)))) OR (((abstract: (postpartum)))) OR (((abstract: (“post-partum”)))) OR (((abstract: ("post partum")))) OR (((abstract: (puerperium))))) OR ((((title: (postnatal)))) OR (((title: ("post natal")))) OR (((title: (“post-natal”)))) OR (((title: (postpartum)))) OR (((title: (“post-partum”)))) OR (((title: ("post partum")))) OR (((title: (puerperium)))))) OR (((((IndexTermsFilt: ("Postnatal Period")))))))) OR (((IndexTermsFilt: ("Postpartum Depression")))))
